# Impact of GaAs(100) surface preparation on EQE of AZO/Al_2_O_3_/p-GaAs photovoltaic structures

**DOI:** 10.3762/bjnano.12.48

**Published:** 2021-06-28

**Authors:** Piotr Caban, Rafał Pietruszka, Jarosław Kaszewski, Monika Ożga, Bartłomiej S Witkowski, Krzysztof Kopalko, Piotr Kuźmiuk, Katarzyna Gwóźdź, Ewa Płaczek-Popko, Krystyna Lawniczak-Jablonska, Marek Godlewski

**Affiliations:** 1 Institute of Physics, Polish Academy of Sciences, Aleja Lotników 32/46, PL-02668 Warsaw, Poland; 2 Department of Quantum Technologies, Faculty of Fundamental Problems of Technology, Wrocław University of Science and Technology, Wybrzeże Wyspiańskiego 27, 50-370 Wrocław, Poland

**Keywords:** atomic layer deposition, external quantum efficiency, gallium arsenide, photovoltaics, surface passivation

## Abstract

In order to effectively utilize the photovoltaic properties of gallium arsenide, its surface/interface needs to be properly prepared. In the experiments described here we examined eight different paths of GaAs surface treatment (cleaning, etching, passivation) which resulted in different external quantum efficiency (EQE) values of the tested photovoltaic (PV) cells. Atomic force microscopy (AFM) and scanning electron microscopy (SEM) examinations were conducted to obtain structural details of the devices. X-ray photoelectron spectroscopy (XPS) with depth profiling was used to examine interface structure and changes in the elemental content and chemical bonds. The photoluminescence (PL) properties and bandgap measurements of the deposited layers were also reported. The highest EQE value was obtained for the samples initially etched with a citric acid-based etchant and, in the last preparation step, either passivated with ammonium sulfide aqueous solution or treated with ammonium hydroxide solution with no final passivation. Subsequent *I*–*V* measurements, however, confirmed that from these samples, only the sulfur-passivated ones provided the highest current density. The tested devices were fabricated by using the ALD method.

## Introduction

The atomic layer deposition (ALD) method is used for silicon passivation in photovoltaics. In recent years we proposed the usage of ALD for the construction of simplified Si-based cells [1]. Once zinc oxide (ZnO) nanorods were employed as a 3D top electrode, 14% of efficiency was reached [2]. Consequently, we also turned out our attention towards gallium arsenide as a substrate/absorber for solar cells. The first results of the experiments made us aware of the potential and possible fields for improvement of ZnO/GaAs-based structures [3]. Due preparation of the substrate, seems to be one of the most important aspects here as the oxide-based states of the GaAs surface are known to adversely affect device performance. Thus, in this present work we focus on the methods to limit their role.

Reactivity of the surface of gallium arsenide is a known and intensively studied phenomenon in semiconductor industry [4]. The air exposure of GaAs surface results in an immediate appearance of different oxides of various compositions (e.g., AsO, As_2_O, As_2_O_3_, GaO, Ga_2_O, Ga_2_O_3_, GaAsO_3_, and GaAsO_4_) as well as of elemental arsenic [5–7]. The presence of an amorphous film of native oxides gives rise to midgap surface states in GaAs [8] which results in Fermi-level pinning [9]. Due to a high surface-related recombination velocity, a decrease in the photoluminescence (PL) of the semiconductor is also observed [7]. These phenomena have strong and negative impact on the performance of GaAs-based microelectronic and optoelectronic devices [10–11]. Therefore, in order to take advantage of the properties of gallium arsenide [12], its interface with a dielectric or other semiconductor partner must be carefully prepared. This can be obtained either by the removal of the native oxide layer followed by an adequate surface passivation technique [13] and/or by a proper choice of the dielectric and its deposition method. Regarding the dielectric, the most common ones are aluminum oxide (Al_2_O_3_) and hafnium dioxide (HfO_2_) for which the preferable deposition method is ALD [14–15].

Removal of native oxide layer and protection of such an obtained surface can be done in many ways. In the case of wet-etching techniques, the most popular GaAs native oxide etchants are based on acidic and basic solutions. In order to etch the oxide, one can treat the surface with an acidic/base aqueous solution (e.g., HCl/H_2_O, NH_4_OH/H_2_O) [16]. If not only the oxide layer but also the suboxide layer of GaAs need to be etched, different methods can be utilized which combine both processes: semiconductor surface oxidation and etching. In this case, also acidic and basic aqueous solutions are used with the addition of an oxidizer – usually hydrogen peroxide (H_2_O_2_). Such an etchant is able to turn GaAs into oxide and dissolve the created oxides “at the same time”. The popular etchants are H_2_SO_4_/H_2_O_2_/H_2_O, NH_4_OH/H_2_O_2_/H_2_O, and citric acid (CA)-based etchants – CA/H_2_O_2_/H_2_O [17–19].

Many protocols for etching solutions are known. Depending on the requirements (e.g., etched compound – oxide or semiconductor, etching profiles, etching rate, ability to remove contaminants – heavy metals, and crystallographic orientation of the substrate) one can use a suitable solution [20–21].

The removal of native oxides by either one of the methods described provides only a partial/interim solution of the problem, as the naked surface of gallium arsenide is afresh vulnerable to quick oxidation when exposed to air or stagnant water [22] (in contrast to running and deoxidized water) [23–25]. Therefore, one should limit the contact time of the GaAs substrate with air/water to an absolute minimum. The attempts that were made to either stop or at least reduce this phenomenon led to the use of surface passivation techniques. One of the well-known and effective methods to protect the surface of GaAs is based on chalcogenide (sulfides or selenides) passivation [7]. In particular, sulfur-containing solutions of compounds such as P_2_S_5_, (NH_4_)_2_S, and Na_2_S perform an effective passivation that substantially reduces further oxidation. In general, the protection mechanism consists in filling As and Ga dangling bonds with adsorbed S atoms, such that covalent bonds (e.g., S–S, As–S, and Ga–S) are observed [26–29]. As a result, the energy of the surface states is changed such that they no longer work as charge traps [30]. This approach has already proven to be a method that improves optical and electrical properties of gallium arsenide-based devices [31–33]. In this work we examine the influence of the surface treatment of GaAs (cleaning, etching, and passivation) on the external quantum efficiency (EQE) results of the AZO/Al_2_O_3_/p-GaAs PV structures (in which AZO stands for aluminum-doped zinc oxide).

## Experimental

### GaAs surface treatment

**Figure 1:**
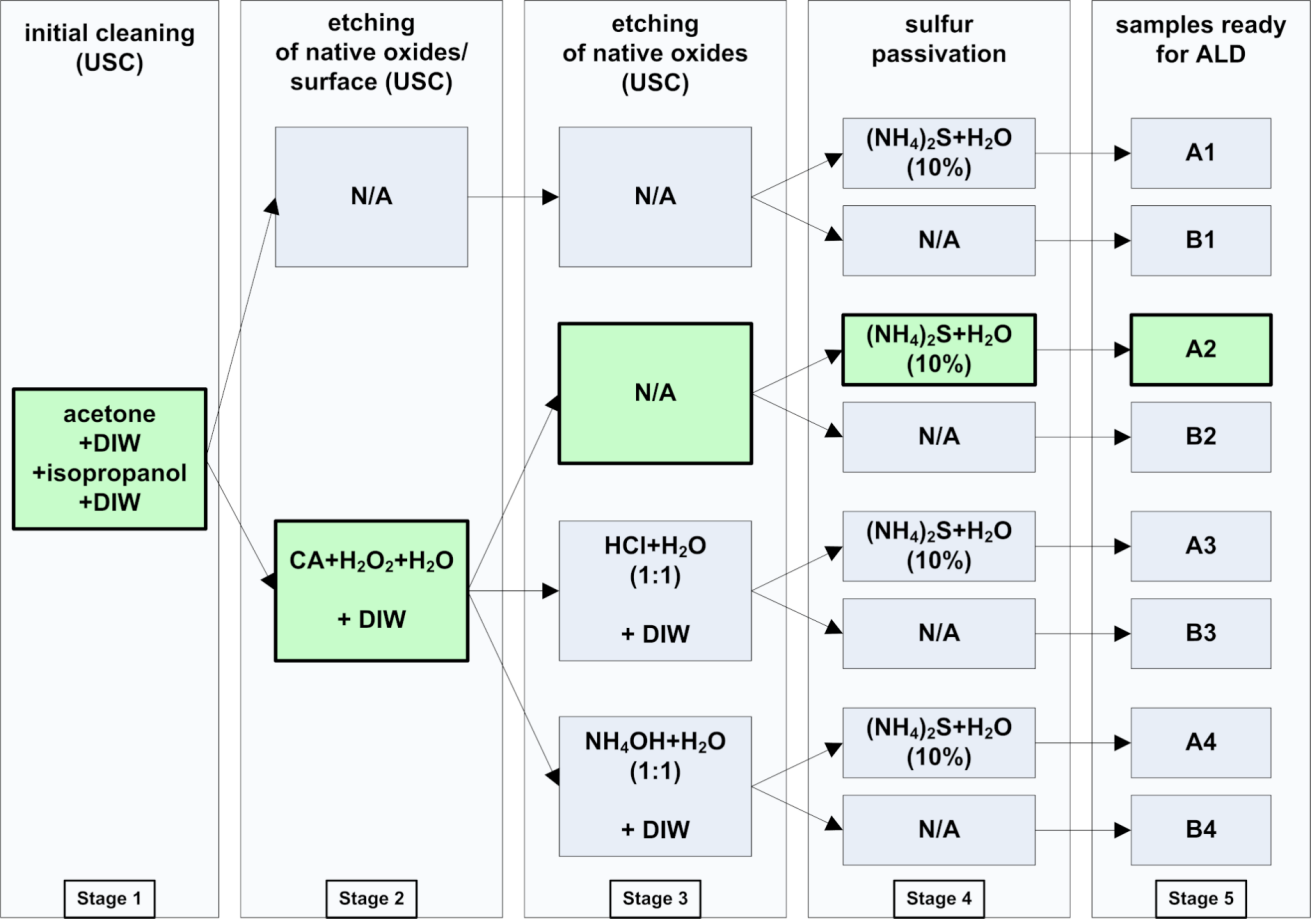
Detailed sequence of GaAs surface-processing steps before the application of the ALD process (the preparation path resulting in the best EQE was highlighted).

We used lightly Zn-doped GaAs single-crystal (100) wafer (*p* = 6.8 × 10^16^ cm^−3^, ρ = 3.2 × 10^−1^ Ω·cm, μ ≤ 225 cm^2^/Vs, *d* = 400 μm) (fabricated at the Institute of Electronic Materials Technology, ITME) as the substrate. Before starting the experiment, the substrate was equipped with an e-beam evaporated (Kurt J. Lesker PVD75) and annealed Au/Zn/Au bottom ohmic contact, which was protected with Kapton tape before further processing steps. In total, eight GaAs samples, denoted as A1–A4 and B1–B4, were fabricated and they differed in terms of the method used for surface preparation. For half of these samples (i.e., the “B” group) the surface was cleaned and/or etched in four different ways distinguished by a numerical suffix (1–4). The second half of samples (i.e., the “A” group) were prepared exactly as the ones from the “B” group (ending with the same suffix) but with an extra sulfur-passivation step utilizing a 10% ammonium sulfide (NH_4_)_2_S aqueous solution (SA10). The passivation was conducted within a separate additional stage just before the ALD process. Thus, the numerical suffixes in both groups denote the specific path of surface preparation up to the last step in which the presence/lack of final passivation classifies the sample as belonging to the group A or B, respectively. The detailed preparation steps are also presented in Figure 1. All the aqueous solutions used in the experiments were prepared with deionized water (DIW, 18.2 MΩ·cm at 25 °C).

The surface of the samples was prepared in five main stages. The stage 1 (initial cleaning in an ultrasonic cleaner, USC) was performed at approx. 30 °C (acetone/3 min, DIW/1 min, isopropanol/3 min, DIW/1 min), while stages 3 and 4 were conducted at room temperature (RT). For the etching of native oxide and suboxide gallium arsenide layers, 50 g of citric acid monohydrate (CA) C_6_H_8_O_7_·H_2_O (CAS: 5949-29-1) was dissolved into 50 mL of DIW. Since this is an endothermic reaction, the solution temperature initially dropped to approx. 8 °C. Further stirring for several minutes raised it to room temperature (RT). Just before the etching process, the dissolved CA in DIW was mixed (1:1, v:v) with hydrogen peroxide (30%). The solution temperature was set to 24 °C and kept at this value during the etching process for 2 min in a USC. The etching rate of such a solution, with the previously mentioned conditions, is approx. 40 Å/s (when stirred) according to Otsubo et al. [34], which gives approx. 480 nm of the substrate etched, including a few nanometers of the native oxide film within 2 min (we assume that the previously mentioned stirring and the use of a USC use gives the same or similar etching rates). After etching in a mixture of CA + H_2_O_2_ + H_2_O, the samples were washed in DIW for 1 min in a USC. Prior contact with an oxidizer as well as the subsequent water bath also resulted in an infinitesimal oxide layer reformation on the surface. This oxide layer was, then, removed from the samples denoted with the suffix “3” by using an aqueous solution containing HCl (37%) (HCl/H_2_O, 1:1, v/v) and from the samples with the suffix “4” by using an aqueous solution containing NH_4_OH (25%) (NH_4_OH/H_2_O, 1:1, v:v) during a 5 min bath in an USC for each set, followed by a subsequent DIW rinse (approx. 5 s) in both cases. The end of this stage resulted in four sets of samples prepared in various ways. The application of the SA10 passivation solution for 20 min at RT on half of the samples (and leaving the other half intact) resulted in eight different samples: A1–A4 (SA10-passivated) and B1–B4 (without a final passivation). These parameters were found to be optimal in a similar experiment, albeit on a different III–V semiconductor substrate [35]). In order to avoid passivation degradation, the A1–A4 samples were not rinsed with DIW after the SA10 treatment [23]. The excess of the ammonium sulfide solution was simply removed by N_2_ blow. Each sample set (A and B) was exposed to air after the final preparation step for approx. 30 min before loading them to the ALD reactor chamber.

### Fabrication of the photovoltaic structures

**Figure 2:**
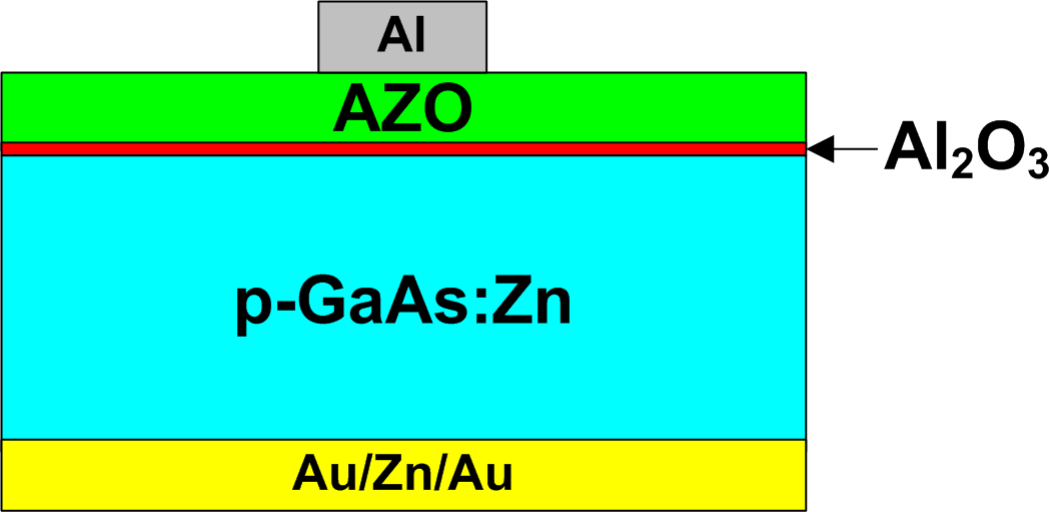
The PV structure used in the experiment.

In order to check how different methods of preparation of the GaAs surface affect the EQE, a simple photovoltaic structure was developed (Figure 2). As an emitter/window, a transparent and thin AZO layer was applied (*d* ≈ 50 nm, *n* ≈ 3.66 × 10^19^ cm^−3^). Beneath it and directly on the recently prepared gallium arsenide surface, we deposited Al_2_O_3_ as the passivation layer in the first few ALD cycles. By applying ALD, we were able to create both Al_2_O_3_ and AZO layers in a single ALD process conducted at 160 °C. Aluminum oxide was deposited within five cycles of (trimethylaluminum/Al(CH_3_)_3_, TMA, CAS:75-24-1) and H_2_O precursor supply while AZO required 10 multi-cycles. Each multi-cycle, in turn, consisted of one aluminum oxide creation cycle (TMA + H_2_O) and 24 cycles of zinc oxide deposition (diethylzinc/Zn(C_2_H_5_)_2_, DEZ, CAS:557-20-0) + H_2_O [2]. In the final fabrication process, a top point contact was deposited (70 nm) by aluminum-target sputtering (Kurt J. Lesker PVD75).

It should also be underscored that the utilized structure was quite simplified and not optimized for the best performance in terms of junction properties, absorber and window layers, doping/thickness, or metallization used.

### Measurement equipment

The morphology of the structures was analyzed using a scanning electron microscope (Hitachi SU-70) with a secondary electron detector operating at 15 kV. The topography of the surface of the layers was analyzed using an atomic force microscope (Bruker Dimension Icon) working in peak-force tapping mode using a ScanAsyst algorithm. A ScanAsyst-AIR probe (Bruker) with a 2 nm tip radius was used for the measurements. Data was analyzed using the NanoScope analysis software (Bruker, version 2.0). The photoluminescence (PL) spectra were taken using a Horiba/Jobin-Yvon Fluorolog-3 spectrofluorometer, equipped with a 750 W xenon lamp as the excitation source and a Hamamatsu R928P photomultiplier. The optical slits used in the measurements were 5 and 1 nm for excitation and emission, respectively. The external quantum efficiency was measured using a Bentham PVE300 photovoltaic system operating with a 75 W xenon lamp and a 100 W quartz halogen lamp. The slit used in the measurements was 0.7 mm which corresponds to an analyzed area spot of 1 mm^2^. In the function of changing the excitation wavelength for 5 nm within the range of 300–950 nm, the number of generated carriers in the examined devices was calculated. Current–voltage light characteristics were collected under standard test conditions (STC) using a PET solar simulator, model #SS100AAA. UV–vis absorbance and reflectance measurements were conducted with the use of a Varian Cary 5000 spectrophotometer equipped with a DRA 2500 integrating sphere accessory. The XPS measurements were performed by applying a Prevac setup equipped with a Scienta R4000 hemispherical analyzer (pass energy of 200 eV) and a monochromatic X-ray tube (Al K_α_ of 1486.7 eV). The full width at half maximum (FWHM) of the 4f_7/2_ Au line measured under the same experimental conditions was 0.6 eV. The O 1s orbital, Al 2p and Zn, Ga and As 3d spin–orbit doublets were measured. The spectra were analyzed using the commercial CASA XPS software package (Casa Software Ltd, version 2.3.17) with Shirley background. The spectra were fitted with a mixed Gaussian–Lorentzian (GL(30)) function. The depth profiling was performed with 2 kV Ar^+^ ions.

## Results and Discussion

### Examination of the devices by SEM and AFM

**Figure 3:**
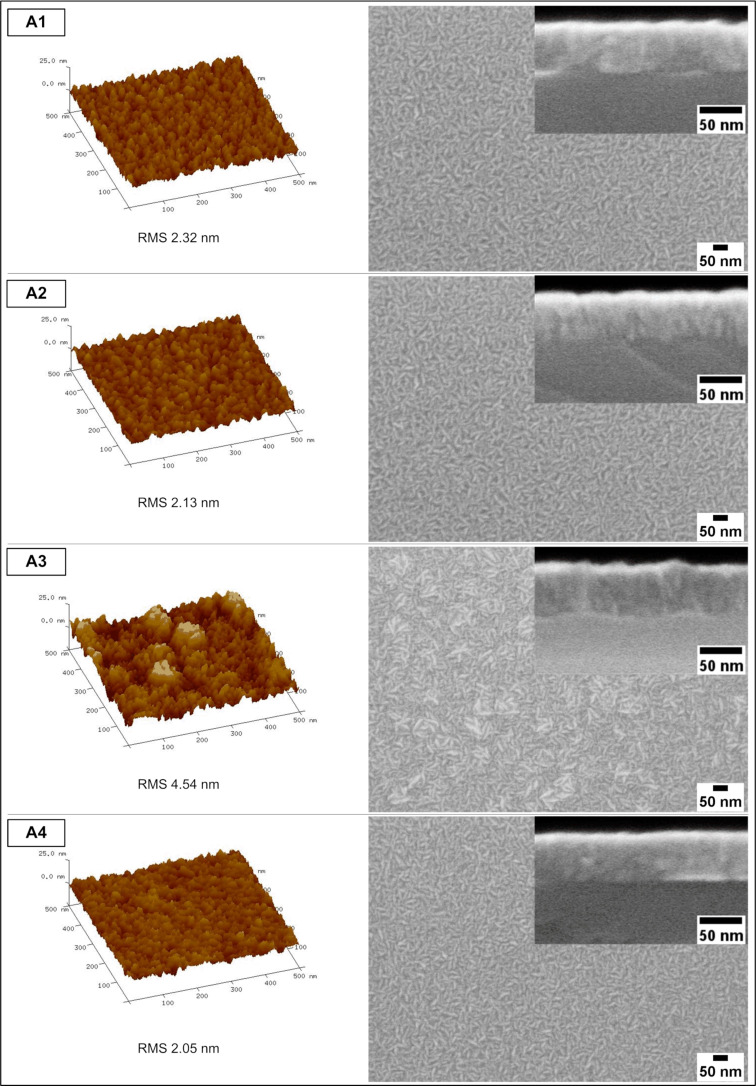
AFM (left panels), SEM top views (right panels), and cross-sections (insets) of A-type samples (the RMS refers to an area of 4 μm^2^).

**Figure 4:**
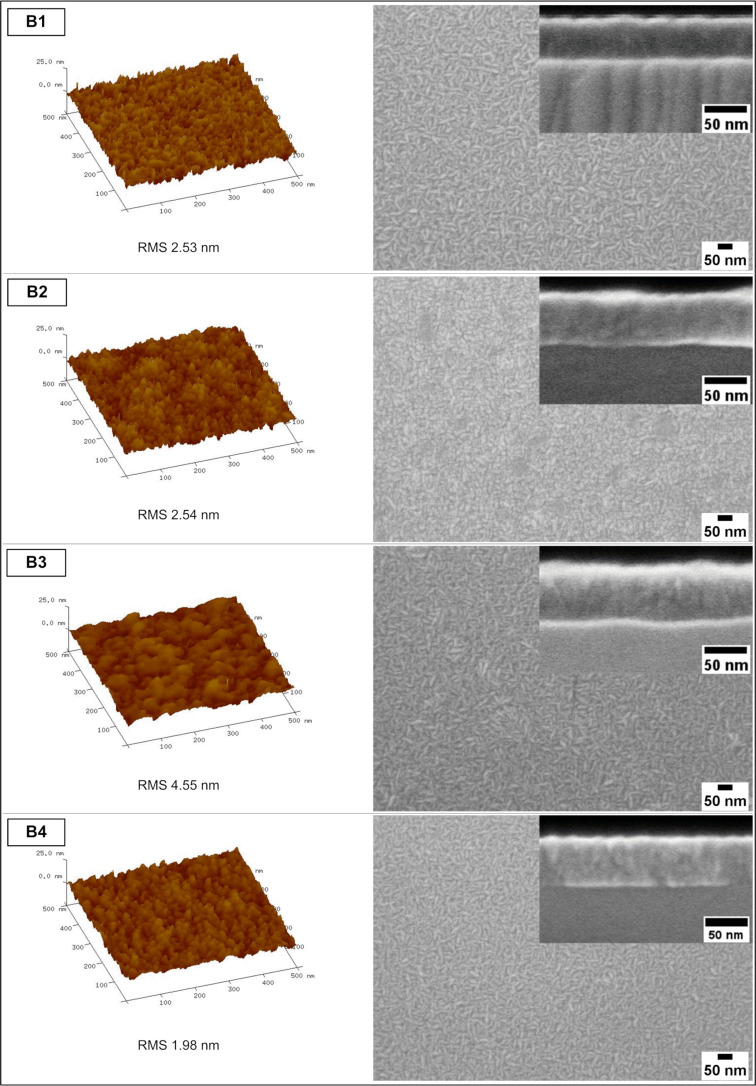
AFM (left panels), SEM top views (right panels), and cross-sections (insets) of B-type samples (the RMS refers to an area of 4 μm^2^).

Figure 3 and Figure 4 present AFM images of the surface of the devices (left side), top-view SEM images (right side), and SEM cross-section images (inset on the right) of the analyzed samples from the A series and B series, respectively.

From the AFM results shown in Figure 3 and Figure 4, one can see that A1, A2, A4, B1, and B4 samples exhibit uniform surfaces by the means of polycrystalline thin film topography. By taking into consideration the roughness of the surfaces (RMS was measured for an area of 4 μm^2^ for every sample), the lowest RMS values are observed in A4 and B4 samples: 2.05 and 1.96 nm, respectively (those samples were treated with the ammonia aqueous solution during stage 3). Conversely, the highest roughness is observed in samples in which the HCl solution was applied (also during stage 3). For A3 and B3 samples we report RMS values of 4.54 and 4.55 nm, respectively. Also, A1 and A2 samples show relatively low roughness, which is likely related to the passivation of the surface with the ammonium sulphide aqueous solution. These values are lower than that of the B1 and B2 samples, in which the SA10 passivation step (stage 4) was intentionally skipped.

The samples prepared using the HCl solution bath (A3, B3) exhibit a predisposition to form crystallized oxide islands at the beginning of the stage of the ALD thin film covering GaAs. Additionally, they show a very rugged surface of GaAs, which is seen on the SEM images of cross-sections and in the RMS values. Conversely, the samples treated with the ammonium hydroxide solution exhibit a high smoothness (i.e., low RMS values). This effect was observed before and is related to the presence of surface hydroxyls promoting a uniform ALD layer growth [36].

The SEM top-view images are in good agreement with the AFM results regarding roughness/surface uniformity. Two kinds of layers can be discriminated: the first layer has a surface suggesting a uniform support (samples A1, A2, A4, B1, and B4) and the second layer is grown on a damaged or on an island-containing substrate (samples A3, B2, and B3). The uniform layers are composed of elongated and fusiform polycrystals, of approx. 50 nm long, which are randomly oriented. The round hills in the samples A3, B2, and B3 are not single crystallites but rather structures uniformly covered with a pattern of AZO crystallites. Worth mentioning are the samples bathed in NH_4_OH solution (A4, B4), as they exhibit a fine-grained uniform surface.

In the cross-sections obtained by SEM (insets in Figure 3 and Figure 4), one can see the approx. 50 nm thick AZO layers for all the samples. The structure of the AZO/GaAs interface is not seen on the images (the intermediate layer was not found between the two layers). One can observe that in the samples A3 and B3 (and more slightly in the samples A2 and B2) a ridged AZO/GaAs interface is present. Conversely, in A4 and B4 samples, the interfaces are exceptionally even in the observed region.

### Characterization of the architecture of the devices by XPS

To understand the interface structure, changes in elemental content, and chemical bonds XPS was performed together with depth profiling. Two representative samples, with the highest (A2) and lowest (B1) EQE, were chosen for detailed studies. The results of elemental content are collected in Table 1 for A2 (left) and B1 (right) samples as a function of the sputtering time. Due a very wide interface we did not convert the sputtering time to nanometers.

**Table 1:** The content of O, Zn, Al, Ga, and As (atom %) in samples A2 (left) and B1 (right) as a function of the sputtering time (min).

A2						B1					
time	O	Zn	Al	Ga	As	time	O	Zn	Al	Ga	As

5	47.9	48	4.1	0	0	5	48.4	48.1	3.5	0	0
15	48.2	48.6	3.2	0	0	15	47.4	48.5	4.1	0	0
25	45.7	50.9	3.4	0	0	25	47.9	48	4.1	0	0
30	45.9	51.1	3	0	0	30	47.9	48.2	3.8	0	0
32	48.1	49.3	2.5	0	0	32	47.1	40.1	4.1	3.6	5.2
34	46.9	49.1	4	0	0	33	44.3	37.2	5.1	6.1	7.3
35	49.9	46.4	3.7	0	0	34	42.9	36.2	5.6	7.3	8
36	47.1	49.3	3.7	0	0	35	41.7	35.9	5.1	7.9	9.4
39	46.5	49.2	4.3	0	0	37	39	31	6.1	11.1	12.8
41	43.5	51.8	3.1	1.6	0	42	32.8	25.9	5.7	17	18.7
43	44.1	51.2	3.4	1.3	0	44	30.5	25.2	6.3	18.7	19.3
45	45.6	50.5	2.9	1.1	0	49	24	20.5	4.1	24.1	27.3
47	46.4	49.4	3.2	1	0	54	15.0	12.4	3.2	34.3	35.1
49	44.8	50	2.7	2.5	0	64	0	1.3	0	52.4	46.2
52	40.4	41.7	4.9	5.3	7.9	—	—	—	—	—	—
55	41.1	41.2	4.4	5.4	7.9	—	—	—	—	—	—
58	37.6	38.9	5.6	8.5	9.4	—	—	—	—	—	—
61	33.6	33.8	5.9	11.9	14.8	—	—	—	—	—	—
66	27.7	30.2	4.5	17.6	20	—	—	—	—	—	—
76	11.1	15.7	3.6	34.4	35.2	—	—	—	—	—	—
86	4	7.1	3.3	42.7	42.9	—	—	—	—	—	—
96	0	4	0	49.7	46.3	—	—	—	—	—	—
106	0	2.8	0	51	46.2	—	—	—	—	—	—

The difference in the width of the interface among the analyzed samples is most striking when comparing the data in Table 1. The substrate was revealed after 96 min of sputtering of sample A2 but already after 64 min of sputtering of sample B1. The changes in elemental concentration appeared after 30 min of sputtering. In the case of A2, first the changes in content of Zn and O were observed and the presence of Ga and As lines was registered only after 52 min. In the case of B1, already after 32 min small amounts of Ga and As were detected. The content of Zn and O in B1 (with only an initial cleaning of the substrate) was 1:1 but in A2 (with the substrate chemically etched and passivated) the ratio between these elements varied between 20 and 50 min of sputtering and an excess of Zn was observed. This evidences a diverse architecture of the interface in the considered samples. The content of Al in the AZO layers was (3.5 ± 0.5)% in both samples. A small increase of Al content was found together with the detection of Ga and As, probably related to the deposition of Al_2_O_3_ as a passivation layer on the substrate in the first few ALD cycles. Therefore, processing of the substrate surface expands (spreads) the interface.

**Figure 5:**
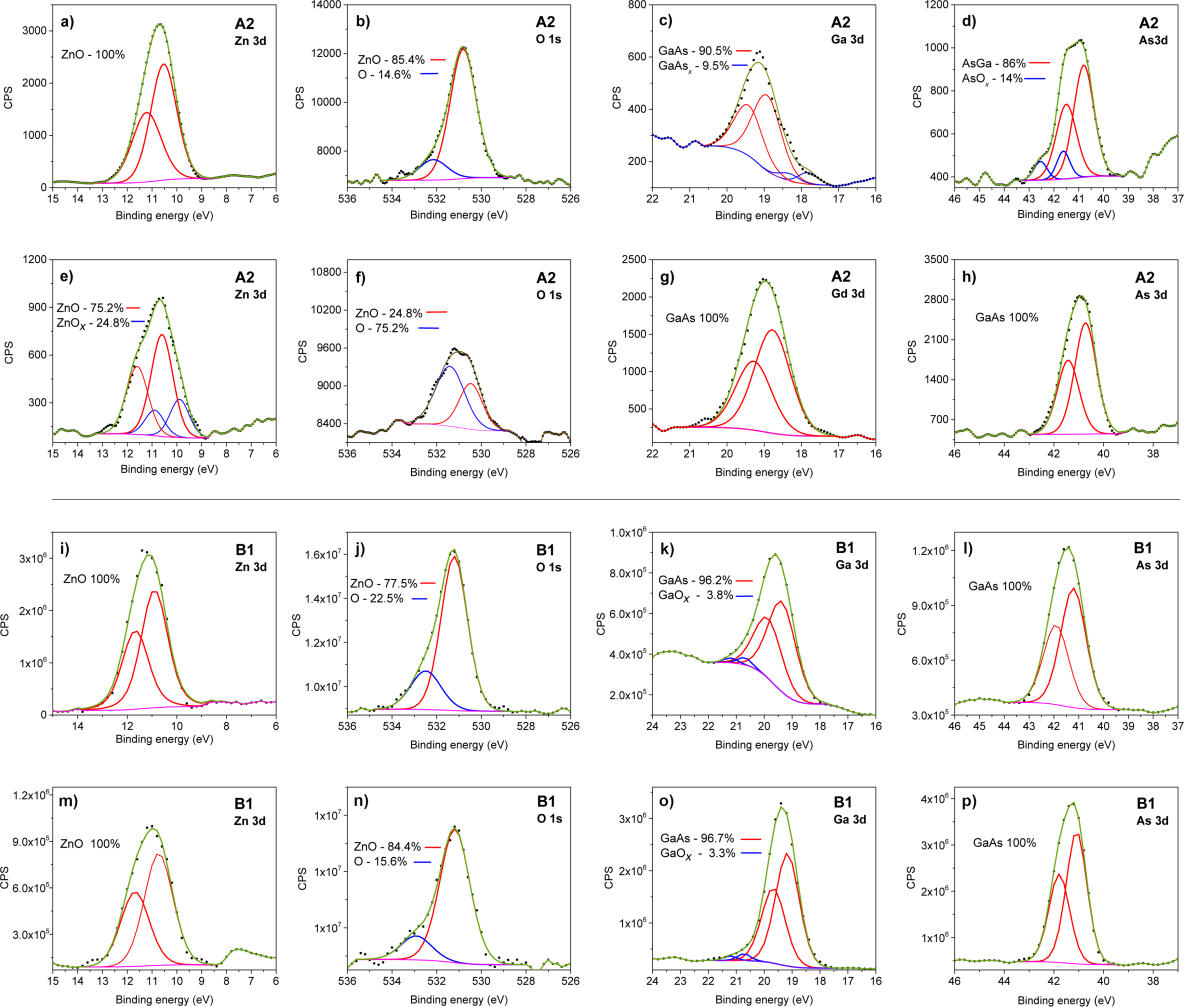
(a–h) A2 spectra after 58 (a–d) and 76 min (e–h) of sputtering. (i–p) B1 spectra after 35 (i–l) and 54 min (m–p) of sputtering. The dots represent experimental data, the red lines denote the spin–orbit or orbit spectra of the main components, and the blue lines denote the additional components. The Shirley-type background is shown in magenta and the dark green line represents the best fit.

To get acquainted with the chemistry of the elements inside the interface, the spectra of Zn, O, Ga, As, and Al at the beginning and close to end of the interface were analyzed and decomposed into possible components. The results are presented in Figure 5a–h for A2 and in Figure 5i–p for B1 samples. No considerable changes in the spectra of the Al sample were detected (not shown). The binding energy (BE) of Al 2p was 74.4 eV as reported for AlOOH [37] and differs from the BE value of Al in AlGaAs (73.7 eV) [38].

When analyzing the shape and BE of the A2 spectra one can notice that the spectra of the elements with a high concentration can be fitted with a single component besides O, which in all cases has two components. The additional component in Ga (Figure 5c) and Zn (Figure 5e) can be ascribed to distorted nearest surroundings due to the lack of As in the case of Ga and O in the case of Zn. The As 3d spectrum has a component with a value higher than that of BE in GaAs which can be ascribed to bonds with oxygen.

The spectra of the elements in the B1 sample do not depend on the concentration of the elements in the interface. The spectra of Zn and As have only one component whereas the spectra of Ga have a small component representing GaO*_x_* and the spectra of oxygen, in both cases, have two components with a slightly different proportion.

### Luminescence of the photovoltaic devices

**Figure 6:**
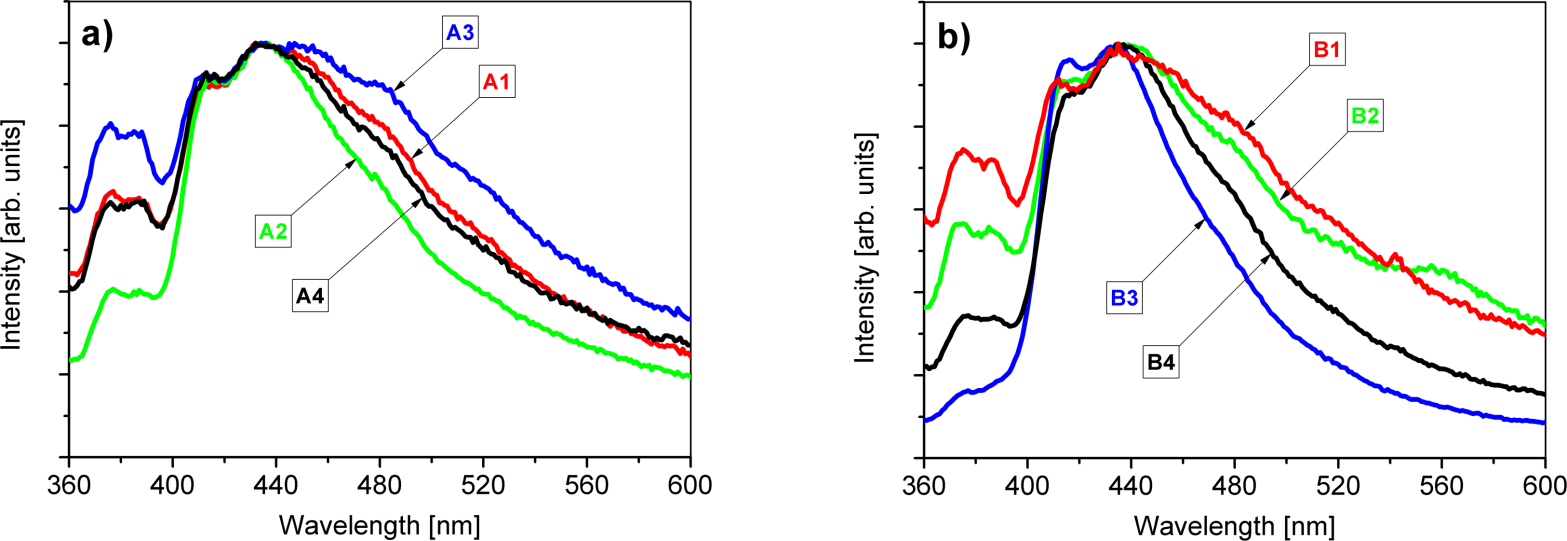
(a) PL spectra (RT) of the samples of the A series. (b) PL spectra (RT) of the samples of the B series.

Figure 6 present the photoluminescence examination results obtained at RT (λ_exc_ = 300 nm) for the SA10-passivated samples (Figure 6a) and for the samples in which the sulfur-passivation step was skipped (Figure 6b).

All spectra were normalized with respect to the feature located at approx. 450 nm. The signals below 400 nm are related to the near-band-edge emission (NBE) in zinc oxide [39]. The luminescence peaks at 413 and 434 nm were proposed before to be resulting from complexes of Al^3+^ ions and V_Zn_ [40]. These complexes resulted from the substitution of Zn^2+^ ions with Al^3+^ ions. The region above 450 nm is ascribed as the reflection of point defects present in the ZnO structure [41]. One can observe a broad band typically present in undoped ZnO [42] with intensity varying depending on the crystallization conditions. Additionally, local maxima may be discriminating the presumable presence of specific defects: 445, 458 (V_Zn_), 480 (Zn_i+_), 516 (V_Zn−_, Zn_O_), and 560 nm (observed only in the B2 sample, possibly due to AsO*_x_* luminescence [43]). The intensities of the defect bands in relation to each other and to the NBE band are associated with the route of AZO growth. The point defects usually observed in ZnO are: oxygen vacancy V_O_, zinc vacancy V_Zn_, interstitial oxygen O_i_, interstitial zinc Zn_i_, and oxygen antisite O_Zn_ [44–45].

The samples in the A series were passivated with SA10 during the preparation of the GaAs substrate, whereas the samples in the B series were not. However, the samples from the A series show a longer and more spread interface between the AZO layer and the GaAs substrate (Table 1). The ratios between the intensities of NBE and DLE change depending on the GaAs surface preparation method. However, there is no clear correlation between the use of SA10 for GaAs passivation and the luminescence of the AZO layer.

### Bandgap size of the AZO layers

Various surface treatments conducted in four different stages (see Figure 1) might induce specific initial growth conditions of proximate layers, possibly resulting in various bandgap sizes of deposited AZO. In order to verify the influence of potential differences of *E*_g_ on the quantum efficiency of the devices, reflectance (diffuse and specular) examinations were conducted. The obtained reflectance spectra were then transformed in order to get the dependence of [*F*(*R*)·*h*ν]^2^ as a function of the energy in eV (where *h* is the Planck’s constant and ν is the frequency of the photon), following the Kubelka–Munk theory and the equation *F*(*R*) = α/*s* = (1 − *R*)^2^/2*R*, where *F*(*R*) is the Kubelka–Munk function, α is the absorption coefficient, *s* is the scattering factor and *R* is the reflectance [46]. After a linear extrapolation within the plot of [*F*(*R*)·*h*ν]^2^ as a function of *h*ν of the relevant area of linear increase, an intersection point with the abscissa was determined for each sample denoting the bandgap size. The results were collected in Table 2.

**Table 2:** Bandgap size of AZO/Al_2_O_3_ layers for the AZO/Al_2_O_3_/p-GaAs and AZO/Al_2_O_3_/glass samples.

architecture	AZO/Al_2_O_3_/p-GaAs								AZO/Al_2_O_3_/glass

sample	A1	A2	A3	A4	B1	B2	B3	B4	AZO/Al_2_O_3_/glass

optical bandgap (direct) *E*_g_ [eV]	3.22	3.24	3.25	3.24	3.23	3.23	3.27	3.23	3.23

To compare the obtained bandgap size results with *E*_g_ of AZO/Al_2_O_3_ grown on a different base, additional measurements were performed with the glass-based sample, where the AZO/Al_2_O_3_ layers were deposited during the same ALD process as for the GaAs-based devices. A glass substrate was chosen as a base for an additional test sample in order to exclude a potential interdiffusion between the substrate and the layers, possibly affecting the properties of the deposited layers, which was likely to happen in the case of the GaAs substrate. Before the ALD deposition process, the glass sample underwent only an initial cleaning (stage 1). In this case the absorbance spectra were collected. The data was then processed in order to get the Tauc’s plot of (α*h*ν)^2^ as a function of the energy in eV, following the equation: (α*h*ν)^2^ = *B*(*h*ν – *E*_g_), where α is the energy-dependent absorption coefficient, *B* is the constant and *E*_g_ is the bandgap energy [47]. Within the obtained plot, the relevant area of rapid linear absorbance growth was extrapolated accordingly, pointing the value of 3.23 eV (the crossing point with the abscissa) as the bandgap of the ALD deposited layers (last column of Table 2). The bandgap size obtained from examining the absorbance spectra is consistent with the *E*_g_ values that we got from the reflectance spectra of the GaAs-based devices. It seems that a different substrate type has either a negligible or no influence on the bandgap size of the AZO/Al_2_O_3_ layers deposited during the ALD process described in this paper.

**Figure 7:**
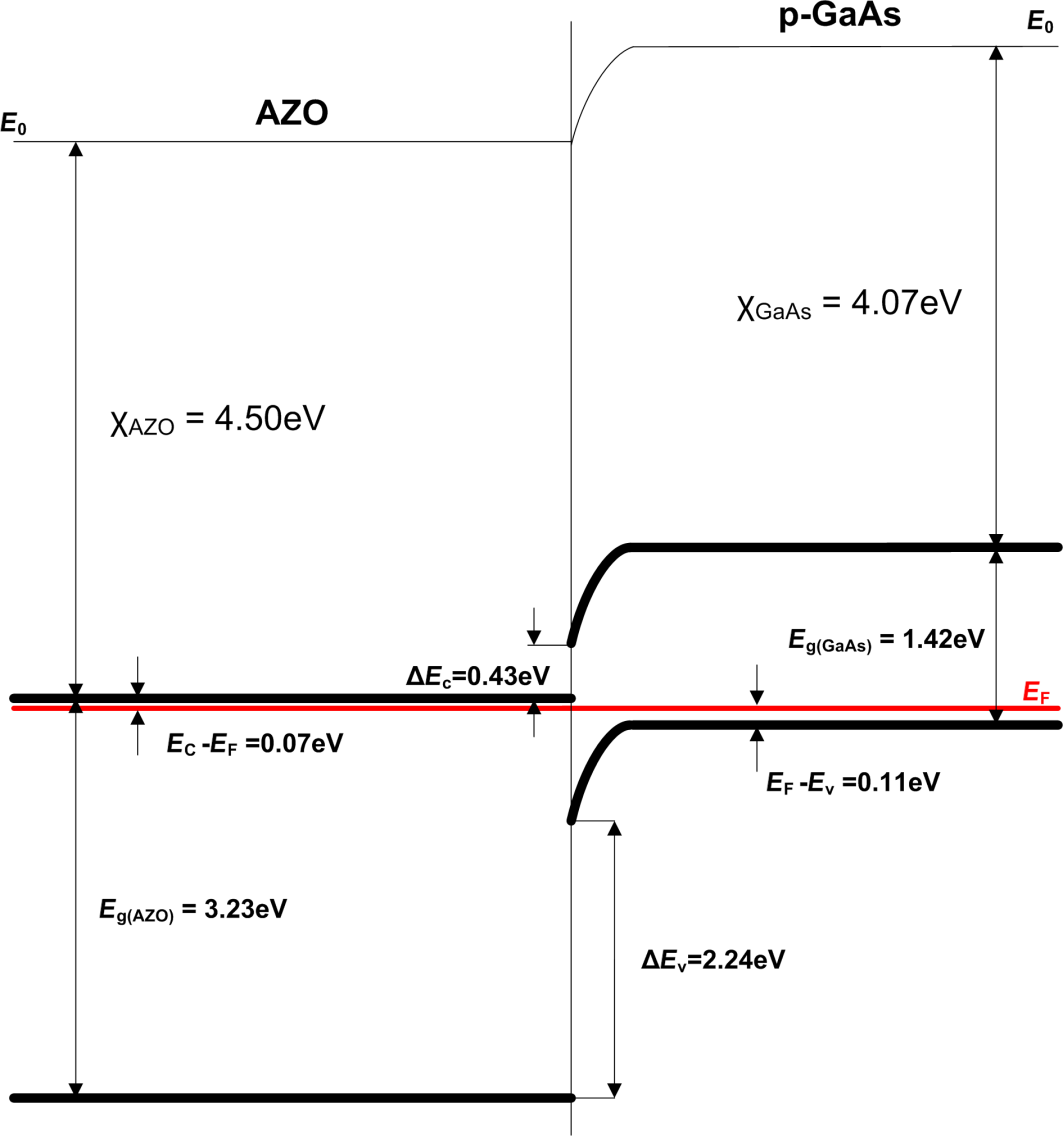
Energy band diagram of the fabricated AZO/Al_2_O_3_/p-GaAs devices.

Knowing the bandgap size of AZO and GaAs, as well as the doping levels and the densities of states in the relevant bands of the semiconductors, we are able to construct the energy diagram of the fabricated devices. The initial illustrative model of the device presented the deposited Al_2_O_3_ passivation coating as a separate dielectric film (Figure 2). However, according to the XPS analysis results (Table 1) one can see that the Al_2_O_3_ deposited within five ALD cycles does not constitute a separate dielectric layer but diffused both the AZO layer and the GaAs substrate instead. In comparison to the AZO layer where the atomic percentage of Al varies between 3–4%, one can find the region in both A2 (in the range of 52–66 min of sputtering) and B1 (in the range of 33–44 min of sputtering) samples where the Al concentration is increased (5–6%); however, it is still quite low and occupies a relatively wide area. Thus it was not included in the energy band diagram (Figure 7) as a separate dielectric layer.

While constructing the diagram for AZO we adopted the following values of electron affinity χ_AZO_ = 4.50 eV [48] and effective density of states in the conduction band *N*_c_ = 2.2 × 10^18^ cm^−3^ [49], while for GaAs we used χ_GaAs_ = 4.07 eV, effective density of states in the valence band *N*_v_ = 7 × 10^18^ cm^−3^ as well as bandgap size of *E*_g(GaAs)_ = 1.42 eV [50]. The bandgap offset values were determined according to the Anderson’s rule for heterojunctions: Δ*E*_c_ = X_AZO_ − X_GaAs_, Δ*E*_v_ = *E*_g(AZO)_ – *E*_g(GaAs)_ + Δ*E*_c_.

### Measurements of the external quantum efficiency

**Figure 8:**
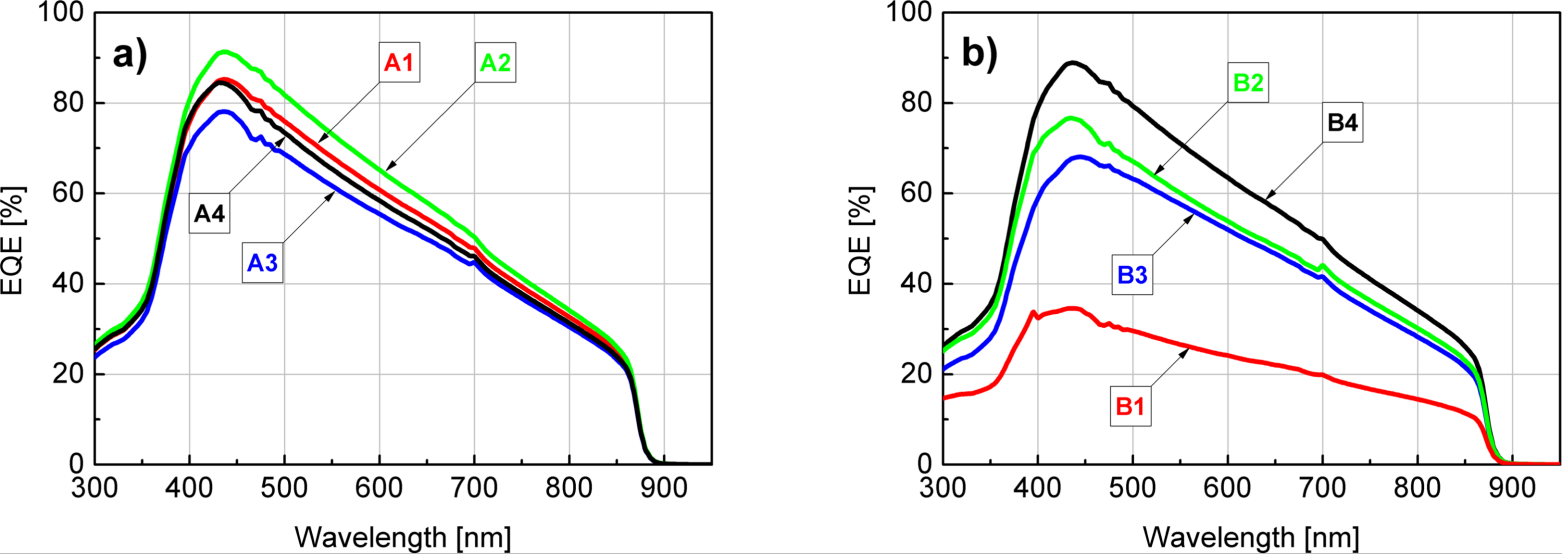
(a) EQE of sulfur-passivated samples (A series). (b) EQE of non-passivated samples (B series).

The EQE characteristics of the examined samples were presented in Figure 8a (A series) and Figure 8b (B series).

The difference between SA10-passivated samples (A series) and their non-passivated equivalent (B series) is clearly seen in Figure 8a and Figure 8b, respectively. Qualitatively, the shape of all features is very similar and they mostly exhibit quantitative differences. In general, we can see that the final passivation step of the GaAs surface considerably increases EQE in almost all cases (with the exception of the A4/B4 set). The highest growth was observed in the case of A1/B1, in which the B1 sample underwent only standard cleaning and the A1 sample is the passivated equivalent. All of the passivated samples exhibit a relatively high EQE compared to the non-passivated samples, with the best curve obtained for the A2 sample in which SA10 was applied directly after the initial cleaning and CA + H_2_O_2_ + H_2_O-based etching (stages 1 and 2). Within the B series, the best result was obtained for the B4 sample which was treated with an NH_4_OH/H_2_O (1:1) aqueous solution during the stage 3 of surface preparation. The lowest external quantum efficiency was found for the B1 sample, which was neither etched nor passivated and underwent only standard cleaning. For both A and B series, the EQE curves exhibit their maxima in a quite narrow range of 430–445 nm. Also, within this interval the relative increase of the quantum efficiency was the highest when the corresponding non-passivated and passivated samples are compared accordingly. The course of all these characteristics is quite similar in the beginning and at the end of the examined spectrum. Starting from 300 nm, and after crossing approx. 350 nm, they rapidly increase and after reaching a maximum value all of them linearly decrease to approx. 20% (approx. 10% in case of B1) at around 875 nm to finally reach zero around 900 nm. The lack of carrier generation at the wavelengths longer than approx. 900 nm stems from reaching the limit of absorption capabilities for the substrate, which for GaAs is 1240.8/*E*_g(GaAs)_ = 1240.8/1.42 eV which gives approx. 874 nm.

For every EQE feature that was collected, its corresponding current density at zero voltage was calculated (Table 3) by multiplying the EQE with AM1.5 Global spectrum (ASTM G-173-03) [51] (converted to spectral photon flux) for each wavelength and integrating the obtained curve according to Equation 1:

[1] $$J_{sc}=-q\int_{\lambda_{1}}^{\lambda_{2}}\Phi (\lambda )\text{EQE}(\lambda )\text{d}\lambda ,$$

where *q* is the charge of electron, Ф(λ) represents the photon flux, and λ_1_, λ_2_ are the limits of the examined spectrum.

**Table 3:** *J*_sc_ calculated from the EQE characteristics.

sample	*J*_sc_ [mA/cm^2^]

A1	17.32 ± 0.46
A2	18.47 ± 0.06
A3	15.88 ± 1.66
A4	16.76 ± 0.35
B1	7.09 ± 1.03
B2	15.58 ± 1.93
B3	14.60 ± 0.96
B4	18.07 ± 0.08

A comparison between the corresponding curve pairs (i.e. A*x* and B*x*), gives more information about the influence of sulfur passivation on the external quantum efficiency for every main path of surface preparation (Figure 8a and Figure 8b). A considerable difference that concerns the first pair of samples (i.e., with suffix “1”) suggests that the existence of native oxides dramatically reduces the EQE of the device and a substantial improvement is instantly obtained after the sulfur passivation is applied. The difference between passivated and non-passivated samples in all the other cases is much smaller. In A2&B2 as well as A3&B3 set, the EQE is further improved if the sulfur passivation step follows the etching process. The cleaning/etching and passivation path, which results in samples with suffix “2”, gives better results than those for the samples ending with suffix “3”, not only for passivated (A2 vs A3), but also for non-passivated samples (B2 vs B3) compared accordingly. In these cases, the acidic bath was applied on the samples before the final passivation. According to the literature, such treatment leaves the As-rich surface with elemental As which has low solubility in acids [52]. Thus, the final treatment with the ammonium sulfide solution should result in the passivation of such surfaces with prevalence of As–S created bonds and lack of (or only a few) Ga–S bonds [13,33,53]. In the case of A4&B4 in which the samples (B4) underwent a final bath in the NH_4_OH/H_2_O (1:1) solution, a subsequent sulfur passivation (A4) seems to have a slight detrimental effect on the EQE. Unlike other samples sets, B4 exhibits higher EQE than the A4 set; however, the difference is not significant. Moreover, B4 features an EQE almost as high as that of A2. As B4 was not SA10-passivated, gaining the second-best EQE result has a different background. We ascribe it to a low interface state density that can be achieved best when Al_2_O_3_ is grown on a GaAs surface previously treated with ammonium hydroxide [36]. The surface preparation in a NH_4_OH/H_2_O (1:1) solution results in a almost stoichiometric Ga/As ratio ranging from 0.84 to 0.94 [36] and even close to 0.99 according to Baca and Ashby [52]. It is also known that NH_4_OH surface treatment leaves it rich in hydroxy groups which facilitate the ALD growth process [36,54–55]. In our opinion, all of the above factors contributed to the relatively high EQE of the B4 sample.

### Current–voltage characteristics

**Figure 9:**
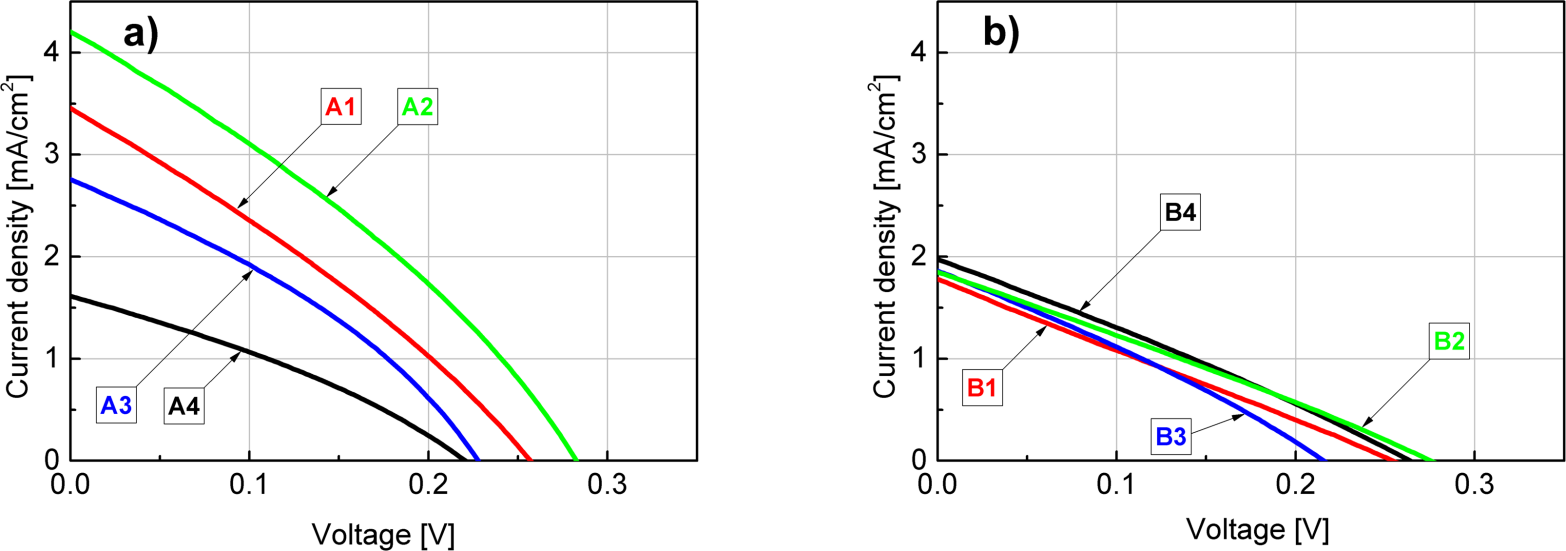
*I*–*V* characteristics of the measured samples of A series (a) and B series (b).

Current–voltage characteristics are presented in Figure 9 for the samples A1–A4 (Figure 9a) and B1–B4 (Figure 9b). The corresponding values of PV parameters were collected in Table 4.

**Table 4:** Photovoltaic parameters of the fabricated AZO/Al_2_O_3_/p-GaAs devices (four samples per architecture).

sample	*V*_oc_ [V]	*J*_sc_ [mA/cm^2^]	PCE [%]	FF [%]	active area [cm^2^]

A1	0.257 ± 0.003	3.45 ± 0.44	0.261 ± 0.039	29.4 ± 0.4	0.16
A2	0.283 ± 0.007	4.21 ± 0.31	0.373 ± 0.023	31.4 ± 0.4	0.17
A3	0.228 ± 0.002	2.76 ± 0.11	0.210 ± 0.001	33.4 ± 0.4	0.18
A4	0.221 ± 0.005	1.61 ± 0.18	0.112 ± 0.018	31.5 ± 0.9	0.21
B1	0.254 ± 0.024	1.78 ± 0.08	0.114 ± 0.016	25.3 ± 0.9	0.15
B2	0.276 ± 0.004	1.85 ± 0.32	0.136 ± 0.024	26.9 ± 0.4	0.24
B3	0.216 ± 0.020	1.86 ± 0.10	0.114 ± 0.006	28.5 ± 0.4	0.20
B4	0.264 ± 0.001	1.98 ± 0.37	0.143 ± 0.027	27.3 ± 0.4	0.20

First it can be noticed that the values of current density for particular samples are substantially lower than the corresponding ones obtained from the EQE analysis. This phenomenon is common, although the mentioned differences are typically lower. In our opinion this particular kind of discrepancy (*J*_sc_ values obtained from EQE higher than those from *I*–*V* measurements) most likely results from the existence of a barrier for the photocurrent, which is effective for a high current density when the sample is illuminated by the solar spectrum. However, a small current density of the quantum efficiency measurement is able to pass the barrier [56]. Another possible reason for the above mentioned discrepancies, which may stem from obvious differences between local EQE as opposed to *I*–*V* measurements of the whole device, is the edge shunt phenomenon as it may be responsible for the majority of the total loss mechanism in the PV device [57]. The impact of other factors, such as a limited spectrum that was examined (300–950 nm) and differences in the spectrum resolution step (5 nm used for EQE measurement as opposed to 0.5–1 nm utilized for the AM1.5 Global spectrum data) should be negligible. Furthermore, we observe that most of the sulfur-passivated devices have a relatively higher current density than the non-passivated ones. Also, A-type samples exhibit a slightly higher fill factor (FF) than the B-type ones (Table 4). However, by comparing the *I*–*V* curves of the A2 and B4 samples that had the highest EQE, we can see that only the A2-originated one is the most effective in this collation, especially regarding *J*_sc_ (Figure 9a and Figure 9b). In this case, the B4 device exhibits a much lower value of current density, comparable to other B-type samples. Also, in the case of the A4 sample, despite the passivation process that was applied, its *I*–*V* parameters are close to those of the B series. It is supposed that, apart from the above mentioned loss phenomena, the lowest RMS of these samples (see Figure 3 and Figure 4) and, thus, the likely increased reflectance is an additional factor contributing to a relatively low current density of the B4 and A4 devices.

## Conclusion

In the present work the influence of GaAs surface preparation on the EQE of simple PV devices was examined. From eight paths tested, we initially determined two paths of GaAs surface treatment that were best for a maximum EQE yield of the PV devices fabricated by ALD. In both A2 and B4 cases, native oxides were first intentionally removed by applying CA + H_2_O_2_ + H_2_O etching solution. Then, the A2 surface was passivated in 10% (NH_4_)_2_S aqueous solution and the B4 sample only underwent a NH_4_OH/H_2_O (1:1) bath. In the case of the A2 sample, the surface preparation sequence (i.e., acidic bath (CA + H_2_O_2_ + H_2_O) followed by passivation with ammonium sulfide aqueous solution) appeared to be optimal for the highest EQE yield. In the case of the B4 sample, despite the lack of sulfur passivation, the EQE was almost as high as in the A2 case. The conducted macro analysis (*I*–*V*) reconfirmed that the A2-specific surface treatment is optimal and allows for obtaining the relative best values of short circuit current density and open circuit voltage. However, this was not the case of the B4 sample, in which unlike the EQE, its *I*–*V* curve differed much from that of the A2 sample, showing a much lower *J*_sc_ value in particular. Thus, only the surface preparation method characteristic for the A2 sample appeared to be optimal from the perspective of both EQE and *I*–*V* curves (the relevant path was highlighted in Figure 1). The bandgaps of all AZO layers were very similar: in the range of 3.22–3.27 eV. The samples were measured using AFM and SEM and the value obtained for the AZO layer thickness was approx. 50 nm in the cross-sections. The topography of the surface depends on the substrate preparation method. The lowest roughness (by means of RMS) was found in the samples etched with ammonium hydroxide solution. Also, these samples exhibited even interfaces as shown in the cross-section images. In contrast, the highest RMS values were found in the samples etched with HCl-based solution. Additionally, the interfaces in these samples were very uneven. There is no explicit correlation between the use of sulfur passivation and the morphology of the samples. The interface structure was more spread in the sample etched with CA-based solution and then passivated with ammonium sulfide aqueous solution (A2 sample, highest EQE). The sample in which no etching solution was applied (B1, lowest EQE) was found to exhibit a consolidated interface. The Al_2_O_3_ interlayer was affected by the solutions used for etching as well as by ALD byproducts and, in fact, the aluminum chemical environment was found to be similar as in AlOOH. In the A2 sample, elements near the AZO/GaAs interface strongly changed as a result of the interdiffusion between the layers in the device. Arsenic underwent oxidation, gallium exhibited distortion related with lowered concentration of As in relation to stoichiometric GaAs. Also, the Zn 3d spectrum shows that the structure of ZnO is defected by the depletion of oxygen, which is accompanied by the appearance of a low-energy O 1s component. Conversely, the B1 sample does not show any significant changes in elements surrounding the AZO/GaAs interface. Only the Ga 3d low-energy component shows a slight oxidation of the GaAs substrate. With regard to the photoluminescence examination, there is an observed luminescence characteristic for zinc substituted by aluminum and accompanied by vacancies. The AZO layers are found to be point defected depending on the surface preparation of the substrate. Also, the *I*_NBE_/*I*_DLE_ ratio depends on the GaAs surface etching procedure before the ALD process.
